# Reconstitution of interferon regulatory factor 7 expression restores interferon beta induction in Huh7 cells

**DOI:** 10.1128/jvi.00703-25

**Published:** 2025-05-23

**Authors:** Andreas Betz, Hao-En Huang, Zuguang Gu, Ombretta Colasanti, Teng-Feng Li, Jasper Hesebeck-Brinckmann, Nadine Gillich, Gnimah Eva Gnouamozi, Matthias Schlesner, Florian W. R. Vondran, Stephan Urban, Ralf Bartenschlager, Marco Binder, Volker Lohmann

**Affiliations:** 1 Department of Infectious Diseases, Molecular Virology, Section Virus-host interactions, Heidelberg University 9144, Heidelberg, Germany; 2 Computational Oncology, Molecular Diagnostics Program, National Center for Tumor Diseases (NCT) and German Cancer Research Center (DKFZ), Heidelberg, Germany; 3 DKFZ-HIPO (Heidelberg Center for Personalized Oncology), Heidelberg, Germany; 4 Division of Virus-Associated Carcinogenesis, German Cancer Research Center (DKFZ) 28333, Heidelberg, Germany; 5 Department of Infectious Diseases, Molecular Virology, Heidelberg University 9144, Heidelberg, Germany; 6 Bioinformatics and Omics Data Analysis, German Cancer Research Center (DKFZ) 28333, Heidelberg, Germany; 7 Biomedical Informatics, Data Mining and Data Analytics, Faculty of Applied Computer Science and Medical Faculty, University of Augsburg 26522, Augsburg, Germany; 8 Department of General, Visceral and Transplant Surgery, Hannover Medical School 9177, Hanover, Germany; 9 German Center for Infection Research (DZIF), Hannover-Braunschweig Site, Hanover, Germany; 10 German Center for Infection Research (DZIF), Heidelberg Site 574554, Heidelberg, Germany; 11 Research Group "Dynamics of Early Viral Infection and the Innate Antiviral Response," Division Virus-Associated Carcinogenesis, German Cancer Research Center (DKFZ) 28333, Heidelberg, Germany; University of Michigan Medical School, Ann Arbor, Michigan, USA

**Keywords:** INFB, interferon, IRF3, Huh7, IRF7

## Abstract

Abundance of essential components of double-stranded RNA (dsRNA) recognition and the subsequent interferon (IFN) response vary widely between primary human hepatocytes (PHHs) and commonly used cell culture models based on derivatives of Huh7 cells, supporting replication of all hepatitis viruses. We used RNA sequencing to compare the innate immune response in hepatoma cells with primary cells and non-neoplastic immortalized hepatocytes (PH5CH). Stimulation with the dsRNA analog poly(I:C) in Huh7 and Huh7.5, either by supernatant feeding or by transfection, resulted in an induction of interferon-stimulated genes widely comparable to that of PHH and PH5CH, but Huh7 and Huh7.5 lacked efficient production of IFN-β. We identified interferon regulatory factor 7 (IRF7) as a critical component missing in Huh7-derived cells. Upon reconstitution of IRF7 expression, *IFNB* induction was restored to the levels observed in PHH upon stimulation with poly(I:C). In contrast to PH5CH cells, for which IRF3 was sufficient for full IFN induction, the lack of IRF7 could not be compensated by increased IRF3 expression in Huh7-derived cells. We further found significant *IFNB* induction upon Sendai virus and hepatitis delta virus infections in Huh7.5 cells reconstituted with IRF7, but not in hepatitis A or hepatitis C virus-infected cells, widely representing the characteristics of the IFN response observed in other models and *in vivo*. Our data suggest that the reconstitution of IRF7 expression in Huh7 cells can aid a more physiological analysis of cell intrinsic immune responses to hepatotropic viruses in future studies.

**IMPORTANCE:**

Current *in vitro* studies often rely on Huh7-based hepatoma cells, which, although permissive for hepatitis viruses, lack critical components for double-stranded RNA recognition. We used RNA sequencing to compare the cell intrinsic innate immune responses of hepatoma cells with more authentic cellular models. We discovered that Huh7-derived cells, which are known to show very limited induction of *IFNB* upon pathogen recognition receptor stimulation, lack expression of interferon regulatory factor 7 (IRF7), an essential component for robust type I interferon induction. By reconstituting IRF7, we were able to restore the interferon response to levels observed in primary human hepatocytes. Our study not only identifies a key missing link in the Huh7/Huh7.5 innate immune response but also offers a way to enhance the physiological relevance of these cells in future studies. Our findings pave the way for more accurate modeling of the human hepatic response to viral infections, potentially improving the understanding and management of hepatitis.

## INTRODUCTION

Viral double-stranded RNA (dsRNA) is an important pathogen-associated molecular pattern which is recognized by several pathogen recognition receptors (PRRs), primarily the endosomal Toll-like receptor 3 (TLR3) and the cytosolic RLRs (retinoic-acid inducible gene I [RIG-I]-like receptors), as RIG-I and melanoma differentiation-associated protein 5 (MDA5). The binding of dsRNA to these PRRs activates signaling cascades through the respective adaptor proteins TRIF for TLR3 (1), and MAVS for RLRs (2). By initiating the NF-κB and IRF3/7 (3–5) pathways, PRR stimulation finally results in the expression of type I and type III interferons (IFNs) as well as of interferon-stimulated genes (ISGs) and of pro-inflammatory cytokines (6). Alternatively to viral infection, this innate immune response can be triggered by synthetic dsRNA analogs, such as polyinosinic:polycytidylic acid [poly(I:C)]. If poly(I:C) is administered to the supernatant of cultured cells, clathrin-mediated uptake to the endosomal compartment will restrict innate immune responses to TLR3 exclusively (7). In contrast, transfected poly(I:C) will additionally activate cytoplasmic PRRs (8). The following induction of ISGs and IFN is widely dependent on IRFs, a family of transcription factors. More specifically, the induction of type I IFN, which is ubiquitously inducible and plays a central role in innate immune response, depends on IRF3 and IRF7 (9). While IRF3 is universally expressed, IRF7 is mainly found in lymphoid cells (10), in which it is known to be pivotal for type I IFN induction (11). Despite the low expression levels in most other resting cells, IRF7 can potently be induced by type I IFN (10).

Several cellular models can be employed for studies of the innate immune response to dsRNA in hepatocytes. Patient-derived primary human hepatocytes (PHHs) express all dsRNA-sensing PRRs and represent the most physiologically relevant cell culture system (12–15). Since they are, however, limited in availability, challenging to manipulate, and barely permissive for some hepatotropic viruses, several other hepatic model cell lines are widely used. Immortalized hepatocytes PH5CH show a comparable cell intrinsic immune response to PHH (15), but are poorly infectable with hepatotropic viruses like hepatitis C virus (HCV), drastically limiting potential applications (16). Huh7-based hepatoma cells are in contrast permissive to many hepatotropic and non-hepatotropic viruses (17), thus representing a versatile model for the study of virus interactions with innate immunity. A limitation of this model is the limited cell intrinsic innate immune response. Huh7 cells lack TLR3 expression (15), while benchmark models for innate immune studies, such as PHH and PH5CH, endogenously express all common dsRNA-sensing PRRs (15, 18). The subclone Huh7.5 is also frequently used for virus infection studies, but besides lacking TLR3 expression, it expresses a dominant negative, dysfunctional RIG-I variant (19). Huh7 and Huh7.5 cells have long been regarded as generally IFN incompetent, regardless of the mode of stimulation, due to additional, but not fully understood, limitations. Characterizing and resolving this malfunction of the IFN induction pathway in Huh7-based cells would be a major step toward obtaining a more physiological virus infection model.

In this study, we employed comprehensive RNA sequencing (RNAseq) analysis to examine the dsRNA response in various hepatic cell lines. Notably, PH5CH cells were capable of inducing type I IFN upon treatment with poly(I:C) to a similar extent as PHH, in contrast to Huh7 cells, including the subclone Huh7.5. Once reconstituted with IRF7, Huh7/Huh7.5 cells augmented *IFNB* mRNA and protein levels comparable to PH5CH and PHH. This suggests that IRF7 expression can compensate for the innate deficiencies in *IFNB* induction in Huh7-derived cells, thereby representing an improved liver-based cell culture model which can recapitulate with high fidelity a physiological innate immune sensing while being highly permissive for hepatitis virus infection.

## MATERIALS AND METHODS

### Cell culture

Huh7, Huh7.5, and PH5CH cells were described previously (20–22). Cells were cultivated in complete Dulbecco’s Modified Eagle’s Medium (DMEM; Life Technologies, Darmstadt, Germany) supplemented with 10% fetal calf serum (FCS), non-essential amino acids (Life Technologies, Darmstadt, Germany), 100 U/mL penicillin, and 100 ng/mL streptomycin (Life Technologies) at 37°C and 5% CO_2_. Cell lines with ectopic gene expression were kept under selection pressure with puromycin (2 µg/mL, Sigma-Aldrich, Steinheim, Germany), blasticidin (5 µg/mL, Life-Technologies), hygromycin (350 µg/mL, Life-Technologies), or neomycin (1 mg/mL, Santa Cruz Biotechnology).

### PRR stimulation

Cells were stimulated with high molecular weight poly(I:C) (InvivoGen, California, USA). If not otherwise specified, cells were stimulated via either adding 10 µg/mL poly(I:C) to the supernatant (SN) or transfecting 0.5 µg/mL (TFX) of poly(I:C) with Lipofectamine 2000 (Life Technologies, Karlsruhe, Germany), following the instructions of the manufacturer. According to the manufacturer, this poly(I:C) mix ranges from 1.5 to 8 kb. If not specified otherwise, 4 h after stimulation, the medium was changed to complete DMEM.

### Isolation and culture of PHHs

For isolates obtained from Hannover Medical School, cell isolation was performed using a two-step collagenase perfusion technique as previously reported (23). Briefly, liver specimens were cannulated and flushed with 500 mL pre-warmed (37°C) washing buffer containing 2.5 mM EGTA. Thereafter, recirculating perfusion with 100 mL of a pre-warmed (37°C) digestion buffer containing 0.05% collagenase (Roche P, Mannheim, Germany) was initiated. Upon sufficient digestion, the tissue was mechanically disrupted, and the emerging cell suspension was poured through a gauze-lined funnel followed by centrifugation (50 *g*, 5 min, 4°C). The resulting cell pellet was washed twice using ice-cold phosphate-buffered saline (PBS) (50 g, 5 min, 4°C) and resuspended in William’s medium E (all Biochrom AG, Berlin, Germany) supplemented with 1 µM insulin, 1 µM dexamethasone/fortecortin, 100 U/mL penicillin, 100 µg/mL streptomycin, 1 mM sodium pyruvate, 15 mM HEPES buffer, 4 mM L-glutamine, and 5% FCS. For isolates from PRIMACYT Cell Culture Technology GmbH, PHHs were thawed according to the manufacturer’s instructions. Cell number and viability were determined by the Trypan blue exclusion test. PHHs were plated in six-well plates at 1.5 × 10^6^ cells per well (Hannover Medical School) or 2 × 10^6^ cells per well (PRIMACYT Cell Culture Technology GmbH) and treated with poly(I:C) as described above at day 1 after seeding.

### Generation of cell lines with ectopic gene expression

For the generation of cell lines, lentiviral vectors were generated and used for transduction as described before (24). A total of 5 × 10^6^ 293T cells were seeded in 10 cm dishes and transfected using polyethylenimine and plasmid pWPI (encoding the genes of interest) together with the packaging constructs pMD2.G (vesicular stomatitis virus G protein) and pSPAX2 (HIV gag-pol). Supernatant containing the lentiviral vectors was harvested and filtered through 0.45 µm filters after 48 h and stored at −80°C. A total of 2 × 10^5^ Huh7 or Huh7.5 cells were seeded into a six-well dish and transduced three times with supernatant containing saturating amounts of lentiviral vectors over a period of 72 h. Transduced cells were selected by the addition of antibiotics in the concentrations described in the cell culture section. The plasmids used for generation of lentiviral vectors encoding TLR3 (25), RIG-I (25), MDA5 (25), LGP2 (26), IRF7 (27), and NTCP (28) were described in previous studies. IRF3 was amplified from plasmid encoding IRF3 (27) using primers ATTATAGGCGCGCCGATGGGAACCCCAAAGCCAC and ATCCAACTCACAACGTGGCA and cloned into pWPI at the AscI and SpeI restriction sites.

### CRISPR-Cas9 knockout (KO) of IRF3 and IRF7

DNA oligonucleotides encoding IRF3- and IRF7-specific single guide RNAs (sgRNAs) were cloned into the lentiCRISPRv2 plasmid and used to generate lentiviral vectors as in the previous section. After antibiotic selection, the knockout pool was checked for knockout efficiency by Western blot and seeded as single cells in 96-well plates. After 2 weeks, the cell clusters derived from single clones were expanded, and knockout efficiency in clones was evaluated by Western blot. Oligonucleotide sequences are as follows: for IRF3, CACCGCTGGTGTCGCAGCTGGACC, and for IRF7, CACCGATCCATACCGAGGCAGCGT.

### HCV infection

A total of 1.2 × 10^5^ Huh7.5 cells were seeded in a 12-well plate 1 day before infection. The cells were infected with the JC1 virus stock described before (29) at a multiplicity of infection (MOI) of 1 and diluted in DMEM without FCS. After 4 h of incubation with the virus on a rocking platform shaker, the cell culture medium was changed to complete DMEM with 1.5% dimethyl sulfoxide (DMSO). At the indicated time points, the cells were harvested for RNA extraction.

### Hepatitis A virus (HAV) infection

A total of 1.2 × 10^5^ Huh7.5 cells were seeded in a 12-well plate 1 day before infection. At least 30 min before infection, the medium was changed to the FCS-depleted DMEM. The cells were infected with the HM175/18f HAV strain (kindly provided by Dr. Yuri Kusov, University of Lübeck) at an MOI of 1.5. After 3 h of infection, cells were washed with PBS and added with complete DMEM. At the indicated time points, the cells were harvested for RNA extraction.

### Hepatis D virus (HDV) infection

A total of 1.5 × 10^5^ Huh7.5 cells were seeded in 24-well plates 1 day before infection. A total of 4 µL pJC126 Genotype 1 HDV stock (30) was mixed with 50 µL 40% polyethylene glycol, diluted in 500 µL complete DMEM containing 1.5% DMSO, and added to the cells (MOI = 1). One day after infection, the cells were washed with 1× PBS. At the indicated time points, cells were harvested for RNA extraction.

### Sendai virus (SeV) infection

A total of 1.2 × 10^5^ Huh7.5 cells were seeded in a 12-well plate 1 day before infection. Sendai virus was diluted in 1× PBS with 0.3% bovine serum albumin (BSA) and added to infect the cells with an MOI of 10. Cells were incubated on a rocking platform shaker for 1 h at room temperature. The medium was then changed to complete DMEM and incubated in a cell culture incubator. At the indicated time points, supernatants were harvested for enzyme-linked immunosorbent assay (ELISA), and RNA was extracted from cell lysates.

### HCV replication inhibition assay

Huh7-ET (Luc-ubi-neo/Et) cells and their use as biosensors to quantify the biological activity of IFNs were described before (31, 32). A total of 3 × 10^4^ cells were seeded in 24-well plates, and 100 µL supernatant from poly(I:C) stimulated cells was added 4 h after seeding. HCV replication was measured via luciferase activity 72 h later.

### RNAseq

RNAseq and RNA quality control were performed by the Genomics and Proteomics Core facility of the German Cancer Research Center using the Illumina Hiseq 4000. For sample preparation, 4 × 10^4^ cells were seeded in a 24-well plate and treated with either 50 µg/mL poly(I:C) added to the SN or TFX of 0.5 µg/mL poly(I:C). Total RNA was extracted after 24 h using the NucleoSpin RNA mini kit (Macherey-Nagel, Düren, Germany) following the manufacturer’s instructions. RNA was quantified using OD measurement at 260 nm.

### Transcriptome data analysis

RNAseq reads were mapped in the human reference genome (build 37, version hs37d5) using STAR (version 2.5.2b) using a two-pass alignment. Duplicate reads were marked using sambamba (version 0.6.5) using eight threads and were sorted by position using SAMtools (version 1.6). BAM file indexes were generated using sambamba. Quality control analysis was performed using the SAMtools flagstat command and the RNA-SeQC tool (v.1.1.8.1) with the 1000 Genomes assembly and Gencode 19 gene models. FeatureCounts (version 1.5.1) was used to perform gene-specific read counting over exon features based on the gencode V19 gene model. The quality threshold was set to 255 (which indicates that STAR found a unique alignment). Raw counts were normalized, and differential expression analysis was performed using DESeq2 (version 1.28.1). Heatmaps were visualized using the ComplexHeatmap package (version 2.5.5). Genes with a false discovery rate (FDR) less than 0.01 were considered for further analysis.

### Gene selection

Genes chosen from the RNAseq data set for differential expression analysis were decided based on their prior characterization in literature, with a special focus taken on genes which play an important role in host defense against viral infection of hepatocytes (14, 33).

### Western blot

Cell lysis, SDS-PAGE, and Western blotting were performed as previously described (34). Cells were harvested in 100 µL lysis buffer (50 mM Tris-HCl pH 7.4, 150 mM NaCl, 1% Triton X-100) containing cOmplete protease inhibitor cocktail (Roche). The samples were adjusted to the same protein concentration by performing the Bradford assay using The Protein Assay Dye Reagent Concentrate (Bio-Rad). Proteins were then denatured by addition of 6× Laemmli buffer (200 mM Tris-HCl pH 8.8; 5 mM EDTA; 0.1% [wt/vol] bromophenol blue; 10% [wt/vol] sucrose; 3% [wt/vol] SDS; 2% [vol/vol] β-mercaptoethanol), incubated at 95°C for 5 min. Protein was separated on 10% SDS-PAGE and blotted onto polyvinylidene difluoride (PVDF) membranes. Afterward, membranes were blocked in 5% skim milk in PBS containing 0.15% Tween 20 (PBS-T) for 1 h at room temperature. Incubation with antibodies targeting RIG-I (AdipoGen Life Sciences, 1:1,000), β-actin (Merck, 1:10,000), calnexin (Enzo Life Sciences, 1:1,000), IRF3 (1:1,000, Cell Signaling), and IRF7 (1:1,000, Cell Signaling) took place overnight at 4°C. Membranes were washed three times for 10 min with PBS-T and incubated with anti-mouse (1:10,000) or anti-rabbit (1:5,000) horseradish peroxidase secondary antibodies (Sigma-Aldrich) for 50 min at room temperature. Detection of chemiluminescence proteins was performed using the enhanced chemiluminescent (ECL) Plus Western Blotting Detection System (Revvity Health Sciences Pierce, GE Healthcare) or SuperSignal West Femto Maximum Sensitivity Substrate (Thermo Fisher Scientific) following the manufacturer’s instruction. Signal was measured using the Advance ECL Chemocam Imager (Intas Science Imaging).

### Quantitative reverse transcription polymerase chain reaction (RT-qPCR)

Isolation of total RNA was performed according to the manufacturer’s description using the NucleoSpin RNA kit (Macherey-Nagel, Düren, Germany). cDNA was generated using the High-Capacity cDNA Reverse Transcription Kit (Thermo Fisher Scientific). qPCR was performed as described before (25), using 2× iTaq Universal SYBR Green Supermix (Bio-Rad) according to the manufacturer’s instructions. To quantify viral RNA of HAV, HCV, and Sendai virus, the qPCR analysis was performed using qScript XLT One-Step RT-qPCR ToughMix (Quanta Biosciences) according to the manufacturer’s instructions. All qPCR experiments were performed in technical triplicate, and glyceraldehyde 3-phosphate dehydrogenase (GAPDH) was used as an internal reference gene. The 2-∆∆CT method (35) was applied to determine relative expression. Bio-Rad real-time PCR systems were used for all qPCR experiments. See Table 1 for the primers and probes used in this study.

**TABLE 1:** Sequences of primers and probes used in this study

Primer or probe	Sequence
Primers	
*GAPDH-f*	GAAGGTGAAGGTCGGAGTC
*GAPDH-r*	GAAGATGGTGATGGGATTTC
*IFIT1-f*	GAAGCAGGCAATCACAGAAA
*IFIT1-r*	TGAAACCGACCATAGTGGAA
*IFN-β-f*	ATGACCAACAAGTGTCTCCTCC
*IFN-β-r*	GGAATCCAAGCAAGTTGTAGCTC
*IFN-λ1-f*	TGGATTGCCCATTTTGCGTG
*IFN-λ1-r*	GAGTGACTCTTCCAAGGCGT
*IRF7-f*	GCTGGACGTGACCATCATGTA
*IRF7-r*	GGGCCGTATAGGAACGTGC
*HCV-f*	GGGCATAGAGTGGGTTTATCCA
*HCV-r*	TCTGCGGAACCGGTGAGTA
*HAV-f*	GGTAACAGCGGCGGATATTGG
*HAV-r*	AGTCAATCCACTCAATGCATCCA
*SeV-P-f*	CAAAAGTGAGGGCGAAGGAGAA
*SeV-P-r*	CGCCCAGATCCTGAGATACAGA
*HDV-f*	GCGCCGGCYGGGCAAC (36)
*HDV-r*	TTCCTCTTCGGGTCGGCATG (36)
Probes	
HAV	6-FAM-TGTTAAGACAAAAACCATTCAACGCCGGA-TAMRA
HCV	6-FAM-AAAGGACCCAGTCTTCCCGGCAATT-TAMRA
SeV	TxRd-GCAGAAGCACATGCTGGAAACCTTG-BHQ2
GAPDH	6-VIC-CAAGCTTCCCGTTCTCAGCCT-TAMRA

### Luciferase assay

Cells were seeded in a 24-well plate format in technical triplicate. For harvesting, cells were washed with PBS, lysed by adding 100 µL lysis buffer per well (1% Triton X-100, 10% glycerol, 25 mM glycylglycine, 15 mM MgSO_4_, 4 mM EGTA, 1 mM dithiothreitol [DTT]), and frozen at −80°C. Luciferase activity was measured using a Mithras LB 940 plate luminometer (Berthold Technologies, Freiburg, Germany) after injection of 400 µL assay buffer (25 mM glycylglycine, 15 mM MgSO_4_, 4 mM EGTA, 1 mM DTT, 2 mM ATP, and 15 mM K_2_PO_4_, pH 7.8 supplemented with 70 µL of 0.1 mM luciferin in 25 mM glycylglycine). To assess HCV replication, data were normalized to the 4 h value to correct for differences in input RNA and transfection efficiency. Inhibition of HCV replication was normalized to untreated samples of the same cell line.

### ELISA

For analysis of interferon secretion, supernatant was collected 24 h after stimulation with poly(I:C), unless otherwise stated. Supernatants were not diluted before use. IFN-β secretion was measured using the LumiKine Xpress hIFN-β 2.0 (InvivoGen, CA, USA) according to the producer’s guidelines.

### Statistical analysis

For statistical analysis between two groups, two-tailed paired *t*-tests using the Holm-Sidak method were performed. Significance between more than two groups was tested with two-way analysis of variance (Dunnett’s multiple comparisons test). All tests were performed using GraphPad Prism 8 software (GraphPad Software, Inc., La Jolla, CA). Further details on statistical tests are indicated in figure legends; *, *P* < 0.05; **, *P* < 0.01; ***, *P* < 0.001, ****, *P* < 0.0001.

## RESULTS

### Transcriptomic analysis of different hepatocytes after poly(I:C) stimulation

**Fig 1:**
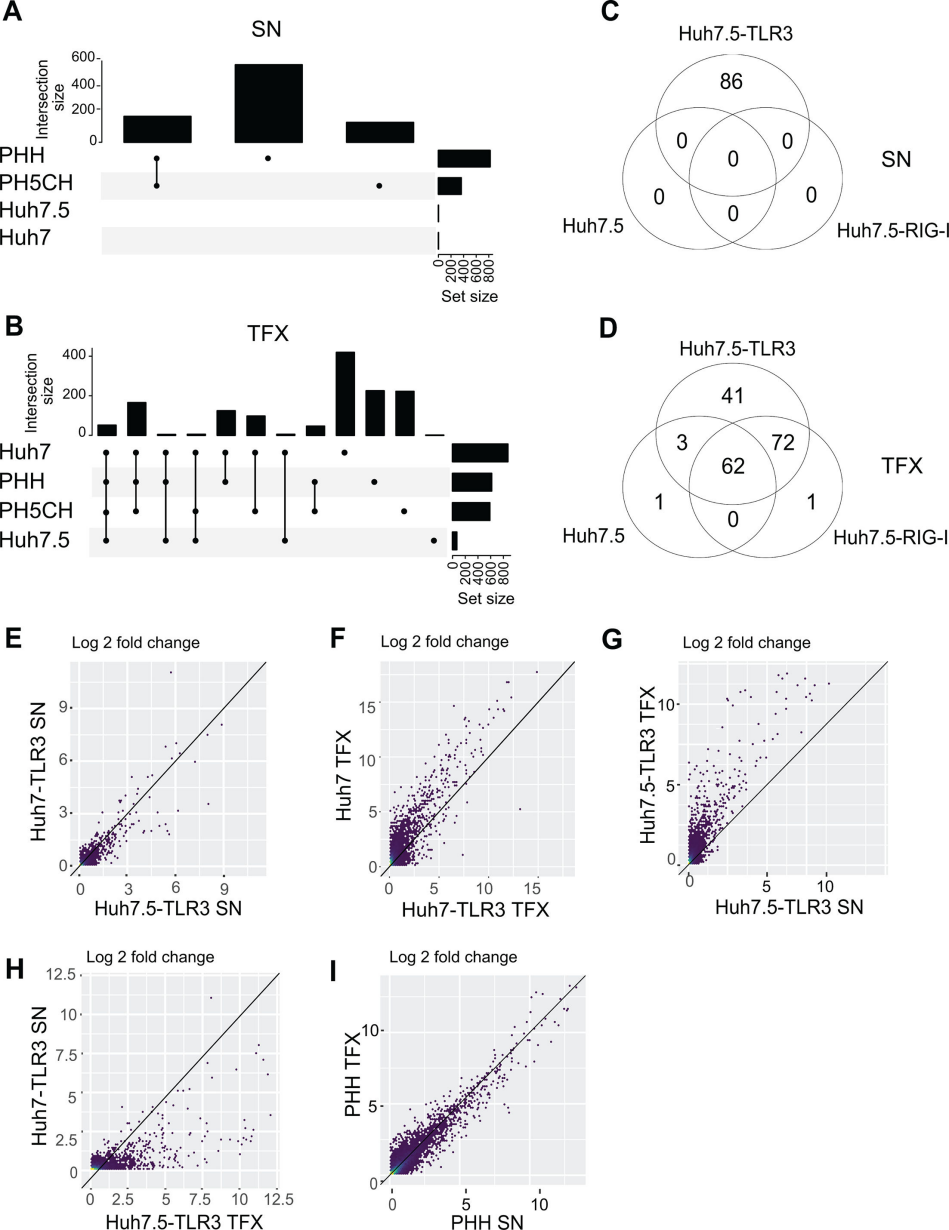
Transcriptomic analysis of different hepatocytes after stimulation with poly(I:C). Cells were stimulated with poly(I:C) either added to the SN (50 µg/mL) or by TFX (0.5 µg/mL). Total RNA was subjected to RNAseq analysis using the Illumina Hiseq 4000 system. (**A, B**) Upset plot of upregulated genes with a log2 fold change (fc) ≥2 compared to unstimulated cells after SN delivery (50 µg/mL) (**A**) or TFX (0.5 µg/mL) (**B**) of poly(I:C). Numbers of upregulated genes for each individual cell line are shown horizontally (set size); numbers of upregulated genes shared by different subsets of cells, marked with dots, which are connected by lines, are given vertically (intersection size). (**C, D**) Gene sets upregulated in Huh7.5 cells, naïve or reconstituted with the indicated PRR shown in a Venn diagram after SN (**C**) or TFX (**D**) of poly(I:C). (E–I) Scatter plot analysis comparing induction levels of individual genes after poly(I:C) stimulation. Only protein-coding genes were considered. Note that PHH and PH5CH endogenously express high levels of TLR3, in contrast to Huh7 cells. All data represent mean values from three biological replicates or four individual donors in the case of PHH.

To better understand the innate immune response to dsRNA and how it is affected by specific expression patterns in different cell lines, we conducted a transcriptome analysis comparing Huh7 and Huh7.5 cells—reconstituted with PRRs—with PHH and PH5CH cells (Fig. 1). The induction of ISGs was quantified by RNAseq upon SN delivery or TFX of high molecular weight poly(I:C), which resulted in a more robust *IFIT1* mRNA induction in the context of ectopic TLR3 expression compared to poly(I:C) with undefined molecular weight (Fig. S1), used in a previous study (25). We chose 24 h after poly(I:C) application to comprehensively include the impact of auto- and paracrine responses by secreted cytokines. SN-applied poly(I:C) induced expression of about 200 genes in PHH and PH5CH cells, including most of the ISGs commonly induced upon dsRNA recognition, with an overall very similar pattern of induction (Fig. 1A; Tables S1 to S9). No genes were induced in the case of both naïve Huh7 variants (Fig. 1A), due to the lack of TLR3 expression (15). In contrast, poly(I:C) TFX resulted in a massive and comparable induction of genes in Huh7, PHH, and PH5CH cells and only a few genes in the case of Huh7.5 cells (Fig. 1B; Tables S1 to S9), due to their reported RIG-I deficiency (19).

Reconstitution of TLR3 or RIG-I expression allowed a comprehensive assessment of the innate immune response to dsRNA in Huh7-based models (25, 26) compared to PHH and PH5CH cells, beyond the established differences in PRR expression. Huh7.5 cells were sensitized toward SN addition of poly(I:C) upon expression of TLR3, but not by RIG-I (Fig. 1C), with an almost identical pattern as in Huh7-TLR3 cells (Fig. 1E). TFX strongly increased the number of upregulated genes in Huh7.5-TLR3 cells compared to SN (Fig. 1C and D), suggesting a more efficient stimulation of TLR3 by this route, as reported (15, 37). In contrast, the response upon TFX was barely impacted by TLR3 in Huh7 cells and likely dominated by endogenous RIG-I (Fig. 1F).

**Fig 2:**
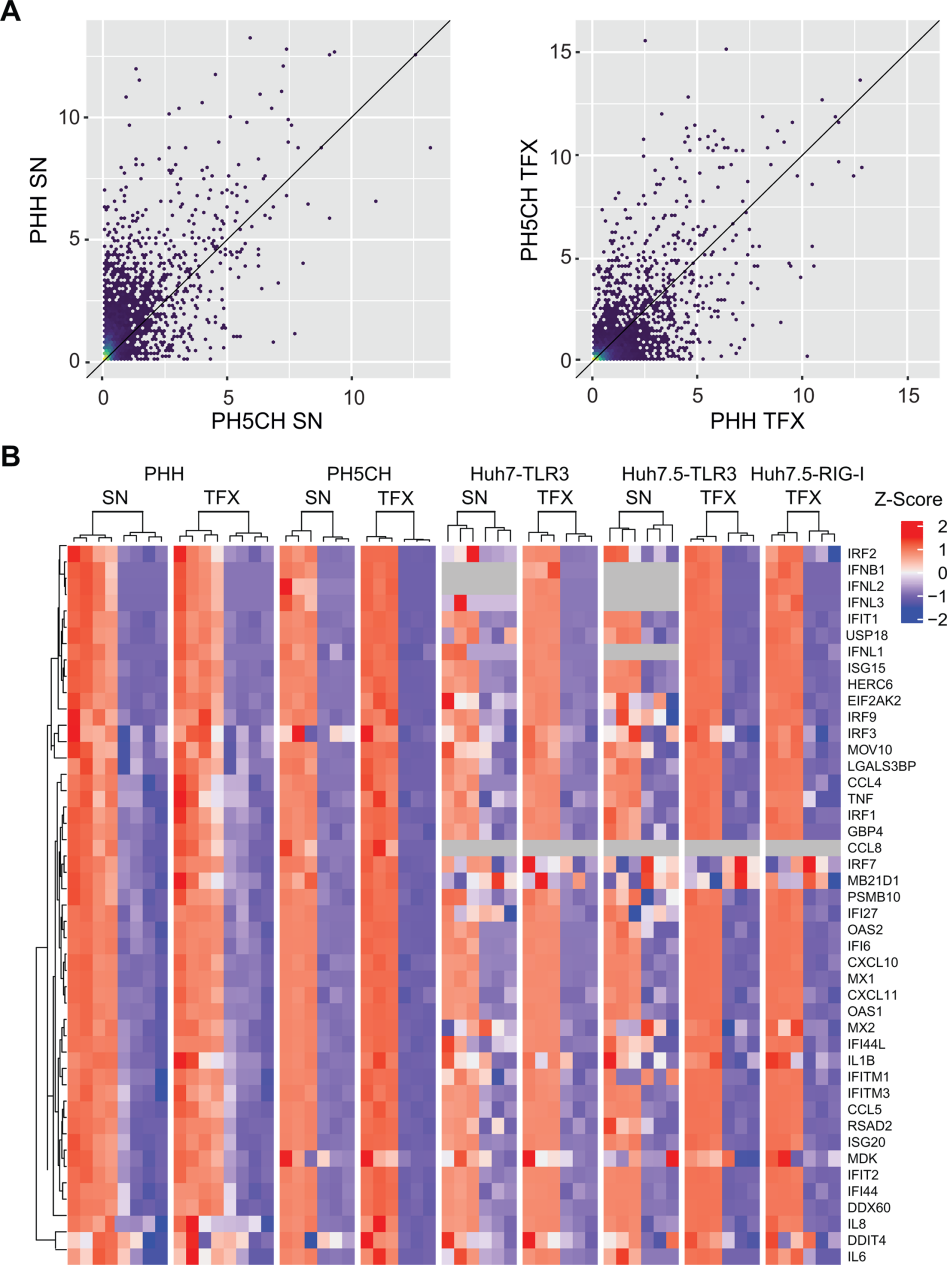
Transcriptomic analysis of individual genes and PRR expression in different hepatocytes after stimulation with poly(I:C). PHH, PH5CH, Huh7, or Huh7.5 cells, either naïve or with ectopically expressed PRR, as indicated, were stimulated with poly(I:C) either delivered to the SN (50 µg/mL) or by TFX (0.5 µg/mL). Six hours after stimulation, the medium was exchanged to DMEM. Twenty-four hours after stimulation, total RNA was extracted and subjected to RNAseq analysis. (**A**) Scatter blot analysis comparing induction levels of individual genes after SN delivery poly(I:C) to PH5CH cells (*x*-axis) or PHH (*y*-axis) (left panel) and comparing TFX of poly(I:C) to PHH (*x*-axis) and PH5CH cells (*y*-axis) (right panel). Log2 fold changes in transcript reads upon stimulation for individual genes or groups of genes are indicated. (**B**) Heatmap showing z-scores of RNAseq data of 36 commonly investigated ISGs. Cell types and stimulation conditions are given on top. The right rows correspond to the non-stimulated replicates, the left rows to the stimulated samples. Note that no IFNA was detectable in any cell type. All data in panels (**A**) and (**B**) represent mean values from three biological replicates or four individual donors in the case of PHH.

The induction pattern of SN and TFX in Huh7.5-TLR3 cells was comparable, but it showed numerous genes that were exclusively upregulated during transfection (Fig. 1G). This pattern also differed when compared to Huh7-TLR3 (Fig. 1H). However, in PHH (Fig. 1I) and PH5CH cells, the response patterns were remarkably similar both in terms of quality and quantity, without any significant distinction based on the poly(I:C) administration method (Fig. 2A).

The sensitivity of RNAseq resulted in high numbers of gene sets significantly upregulated in individual cell lines based on low absolute read numbers with questionable relevance (Fig. 1A and B; Tables S1 to S9). We therefore focused on a collection of commonly investigated ISGs and IFNs (Fig. 2B) (14, 33) to better understand the differences in the PRR-driven response between cell lines and experimental settings. As expected, the entire set of analyzed genes was induced in PHH and PH5CH cells, independent of the mode of poly(I:C) delivery. The same pattern was found upon poly(I:C) TFX to all Huh7-derived cells, except a few genes that were entirely or almost absent and/or non-inducible (e.g., IRF7, CCL8, MDK; Fig. 2B; Tables S1 to S9) and lower induction levels upon supernatant delivery of poly(I:C) in Huh7 cell lines expressing TLR3, compared to PHH and PH5CH (Fig. 2B). Most strikingly, we found no induction of type I and type III IFN transcription upon SN delivery to Huh7 and Huh7.5 cells with reconstituted TLR3, in stark contrast to PH5CH and PHH (Fig. 2B).

### Kinetics of IFN induction in different cell lines of hepatic origin

**Fig 3:**
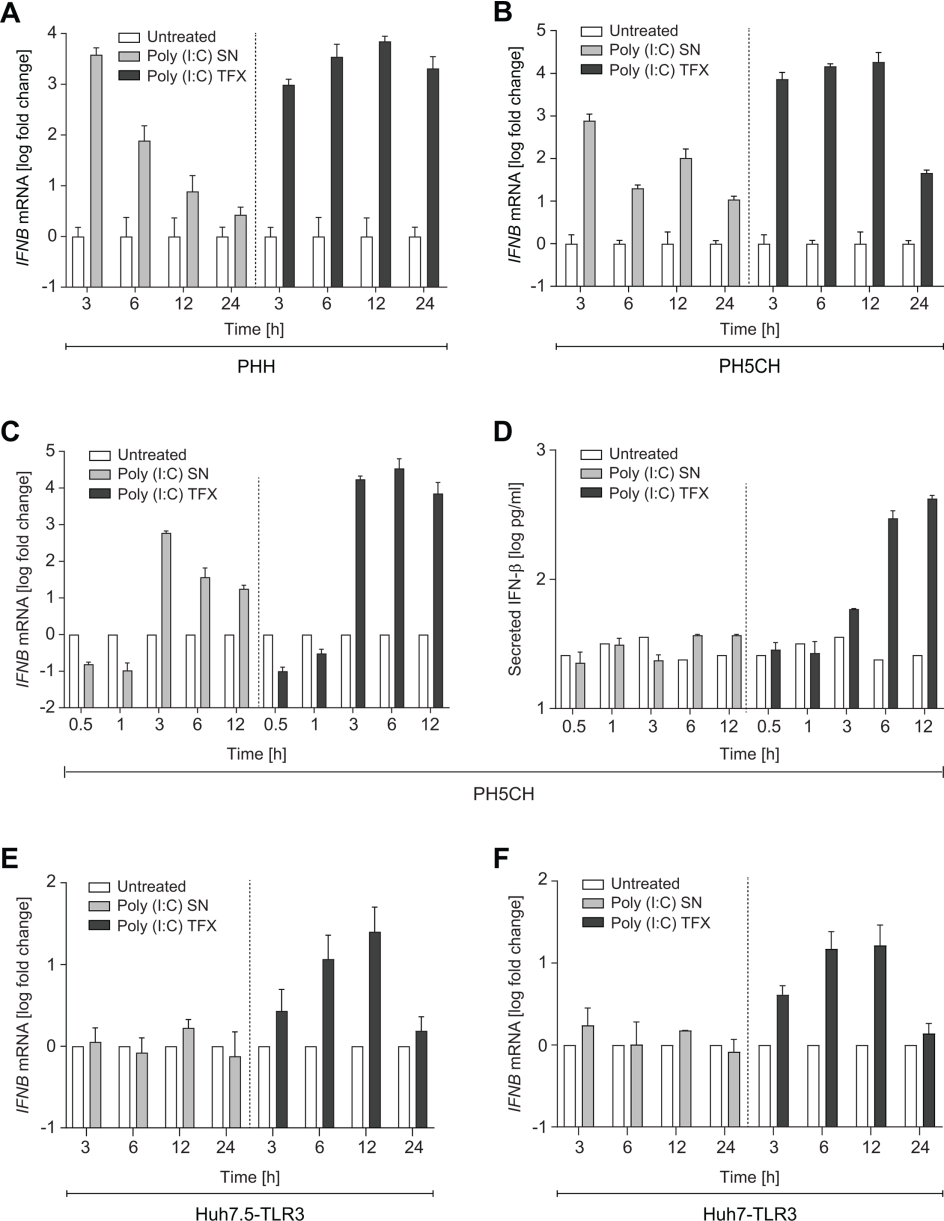
Analysis of *IFNB* induction upon poly(I:C) stimulation. Indicated cells were stimulated with poly(I:C). The dotted line separates SN (10 µg/mL) and TFX (0.5 µg/mL) stimulation. Time course of (A–C**, E, F**) *IFNB* mRNA expression and (**D**) IFN-β secretion upon SN delivery (left) and TFX (right) in PHH (**A**), PH5CH (B–D), Huh7.5-TLR3 (**E**), and Huh7-TLR3 cells (**F**) at the indicated time points. mRNA levels were determined by RT-qPCR. Data are normalized to *GAPDH* and shown as fold relative to the same cells in the absence of poly(I:C). Data represent mean values and SD from technical triplicates of one representative experiment (A–D) or from technical triplicates of two biological replicates (**E, F**). (**D**) For the time course of secreted IFN-β, cell supernatant was harvested and analyzed in technical duplicates by ELISA.

To better understand the observed lack of IFN induction in Huh7-based cells, we compared the time course of *IFNB* expression in PHH, PH5CH, Huh7, and Huh7.5 cells upon poly(I:C) stimulation (Fig. 3). We focused on *IFNB* since no other type I IFN was induced in any of the hepatocytes included in this study (Tables S1 to S9). In PHH and PH5CH, SN delivery of poly(I:C) resulted in a strong but very transient induction of *IFNB* mRNA, peaking already at 3 h after induction, in line with a previous study (13). In contrast, TFX of poly(I:C) induced far stronger and more sustained *IFNB* expression (Fig. 3A and B). We further analyzed earlier time points in PH5CH and verified that *IFNB* mRNA was not detectable prior to 3 h (Fig. 3C). Interestingly, no IFN-β was secreted upon SN-mediated induction, whereas it became detectable at 6 h after TFX of poly(I:C), suggesting that ISG levels determined at 3 h were solely based on direct induction of PRRs, and not on autocrine IFN stimulation (Fig. 3D). In striking contrast, SN feeding of poly(I:C) did not at all induce transcription of *IFNB* mRNA in Huh7.5-TLR3 (Fig. 3E) and in Huh7-TLR3 cells (Fig. 3F). TFX of poly(I:C) resulted in a similar kinetics of *IFNB* induction as in PHH and PH5CH cells, but levels were several orders of magnitude lower.

In summary, we observed potent ISG and IFN induction in PHH and PH5CH cells starting from 3 h post-stimulation. In comparison, *IFNB* induction upon TFX of poly(I:C) was reduced around 3 log-fold in Huh7 and Huh7.5 cells.

### IRF7 rescues *IFNB* induction in Huh7 and Huh7.5 cells

**Fig 4:**
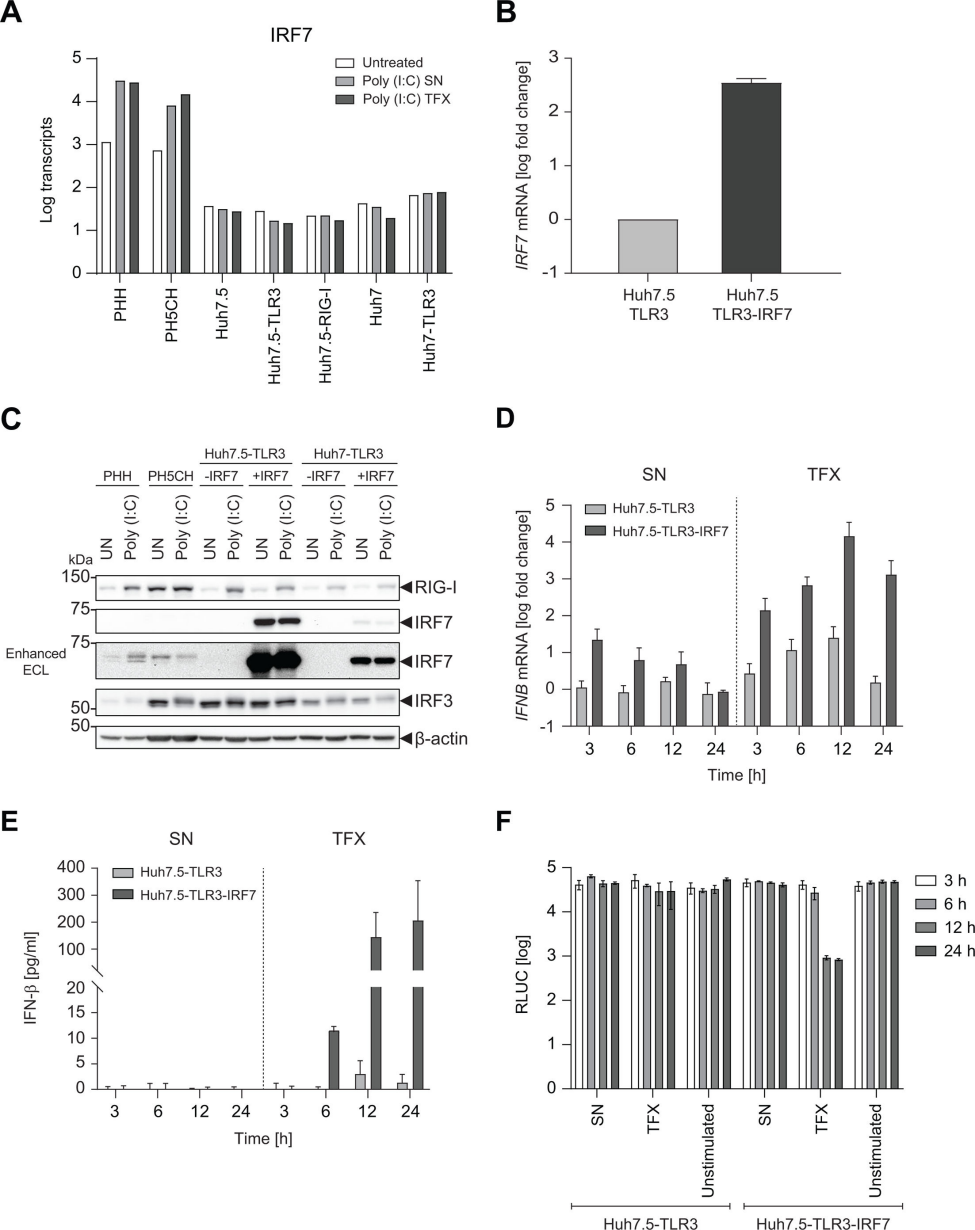
Impact of IRF7 on the IFN response in Huh7.5 cells. (**A**) *IRF7* expression in different hepatic cell lines. Transcript counts were obtained by RNAseq (see Tables S1 to S9). (**B**) Huh7.5 cells were transduced with lentiviral vectors encoding *TLR3* and *IRF7*, as indicated. The level of *IRF7* mRNA was quantified using RT-qPCR. *IRF7* expression was normalized to *GAPDH* and shown as fold relative to the Huh7.5-TLR3 cells. (**C**) Indicated cells were either left untreated (UN) or transfected with 0.5 µg/mL poly(I:C) for 8 h. RIG-I, IRF7, IRF3, and β-actin protein expression were determined by Western blot, using ECL plus substrate. In the case of IRF7, the blot was in addition developed with SuperSignal West Femto Maximum Sensitivity Substrate (Enhanced ECL) to increase sensitivity. Data from one representative of two independent experiments are shown. (D–F) Huh7.5-TLR3 cells were transduced with lentiviral vectors encoding *IRF7*. Cells were stimulated with poly(I:C) either delivered to the SN (10 µg/mL) or by TFX (0.5 µg/mL). At the indicated time points, total RNA was extracted, and mRNA levels of *IFNB* were determined using RT-qPCR (**D**). Data are normalized to *GAPDH* and shown as fold relative to the untreated Huh7.5-TLR3 cells. In addition, secreted IFN-β was quantified in cell supernatant by ELISA in technical duplicates (**E**), and biological activity of secreted IFNs was determined using indicator cells (**F**). Supernatants from indicated cells and conditions were harvested at different time points after poly(I:C) or mock (untreated) stimulation and transferred to Huh7 cells containing a persistent HCV reporter replicon, encoding firefly luciferase (31). Luciferase activity was determined in cell lysates 72 h after supernatant transfer and is shown in relative light units (RLU). Reduced RLU counts indicate inhibition of viral replication by secreted IFNs. (**C**) shows data from one representative experiment. Data represent mean values and SD of technical triplicates from two biological replicates (B, D–F) or mean values from three biological replicates or four individual donors in the case of PHH (**A**).

To identify the reason underlying the robust ISG induction but insufficient IFN induction upon poly(I:C) stimulation, we searched for important components of cell intrinsic innate immune responses consistently missing in Huh7-based cells. IRF7 is a key factor involved in the induction of IFN transcription (38), and therefore appeared as a plausible candidate factor underlying the IFN deficiency in cells originating from Huh7, since it was entirely absent in Huh7 and Huh7.5 cells, but expressed and strongly inducible on mRNA level in PHH and PH5CH (Fig. 4A and 2B; Tables S1 to S9). We therefore reconstituted IRF7 expression by lentiviral transduction in Huh7- and Huh7.5-TLR3 cells (Fig. 4B and C) and in a panel of Huh7.5 cells expressing additional or alternative PRRs (Fig. S2). On the protein level, Huh7 and Huh7.5 expressed comparable amounts of RIG-I and IRF3 as PHH and PH5CH, but indeed entirely lacked IRF7 expression (Fig. 4C). After lentiviral transduction, IRF7 expression was far higher in the different Huh7-derived cell lines even compared to poly(I:C) stimulated PHH and PH5CH and remained unaffected by poly(I:C) stimulation, as expected (Fig. 4C). Still, even such high levels of *IRF7* expression did not cause detectable ISG induction in the absence of poly(I:C) (data not shown).

Functionally, IRF7 expression resulted in a moderate transient induction of *IFNB* mRNA in Huh7.5-TLR3 cells upon SN delivery of poly(I:C), lacking secretion of IFN-β. Importantly, TFX of poly(I:C) induced a massive increase in *IFNB* transcription, followed by substantial secretion of the cytokine (Fig. 4D and E), very similar to PH5CH cells (Fig. 3C and D). The secreted IFN-β also resulted in an increased and more sustained induction of *IFIT1* expression, again comparable to PHH and PH5CH (Fig. S3A, C, and E). Type III IFN induction was overall similar to *IFNB* in PH5CH and PHH (Fig. S3B and D). In Huh7.5-TLR3 cells, IRF7 also restored *IFNL1* mRNA induction upon SN feeding of poly(I:C), but it had only limited impact on *IFNL1* induction upon poly(I:C) transfection, since here, the deficiency was less pronounced compared to *IFNB* (Fig. S3F). Still, the induction of type III IFN mRNAs appeared not to contribute to the antiviral potency of secreted cytokines, since the inhibitory action of supernatants obtained after poly(I:C) stimulation exactly mirrored the quantity of secreted IFN-β (Fig. 4F). Very similar results were obtained in Huh7-TLR3 cells reconstituted with IRF7 (Fig. S4A through C), although they displayed a far lower IRF7 expression level than their Huh7.5 counterparts (Fig. 4C), suggesting that IRF7 abundance was not a limiting factor for the magnitude of effects. Interestingly, despite lower levels of IRF7 expression in Huh7, small amounts of IFN-β were also secreted upon SN feeding with poly(I:C) and not only upon TFX (Fig. S4D), resulting in a detectable antiviral effect (Fig. S4E).

In conclusion, expression of IRF7 restored the ability of Huh7- and Huh7.5-TLR3 expressing cells to produce IFN-β in response to transfected poly(I:C).

### Contribution of IRF7 and IRF3 to *IFNB* induction in PH5CH and Huh7.5 cells

**Fig 5:**
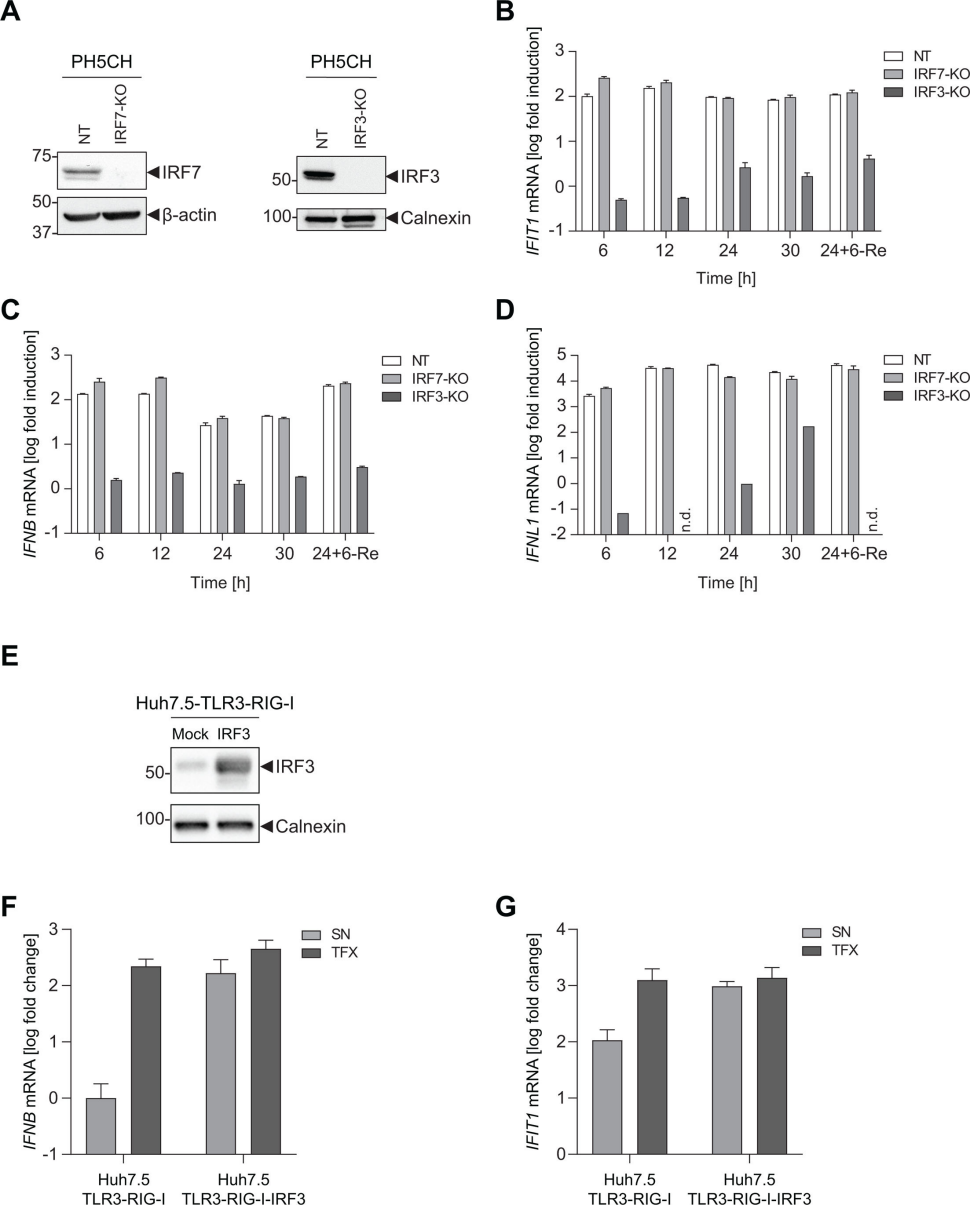
Function of IRF3 and IRF7 in PH5CH and Huh7.5 cells. (**A**) PH5CH cells were transduced with lentiviral vectors encoding CRISPR-Cas9 and sgRNAs targeting *IRF3*, *IRF7,* or a non-targeting control (NT). Knockout efficiency was validated by Western blot and is shown for a single clone. Since *IRF7* expression is mostly absent in resting cells, knockout efficiency was determined 6 h after TFX (0.5 µg/mL) of poly(I:C). (**B**) *IRF3*/*IRF7*-KO were stimulated with poly(I:C) by TFX (0.5 µg/mL). Twenty-four hours after the initial stimulation, one condition was stimulated again by TFX of 0.5 µg/mL poly(I:C) for 6 h (24+6-Re). At the indicated time points, total RNA was extracted, and mRNA levels of (**B**) *IFIT1*, (**C**) *IFNB*, or (**D**) *IFNL1* were determined using RT-qPCR. Data are normalized to *GAPDH* and shown as fold relative to untreated naïve cells. Data represent mean values and SD of technical triplicates from *n* = 4 biological replicates. (B–D) or one representative experiment (**A**). n.d., not detectable. (E–G) Huh7.5 cells stably expressing TLR3 and RIG-I were transduced with lentiviral vectors encoding *IRF3* and selected to obtain cells stably overexpressing IRF3. (**E**) IRF3 expression levels were evaluated by Western blot in comparison to non-transduced cells. (**F, G**) Huh7.5-TLR3-RIG-I cells in the presence and absence of IRF3 overexpression were stimulated with poly(I:C) either delivered to the SN (10 µg/mL) or by TFX (0.5 µg/mL). Twelve hours after treatment, total RNA was extracted, and mRNA levels of (**F**) *IFNB* or (**G**) *IFIT1* were determined using RT-qPCR. Data are normalized to *GAPDH* and shown as fold relative to the untreated naïve cell. Data represent mean values and SD of technical triplicates from two biological replicates (**F, G**) or one representative experiment (**E**).

Our data so far indicated that IRF7 had a substantial role in the induction of *IFNB* after poly(I:C) stimulation in Huh7-derived cells. We next aimed to understand whether IRF7 was equally important in PH5CH cells, and therefore generated IRF7 and IRF3 KO cells by CRISPR-Cas9 (Fig. 5A). KO of IRF3 entirely abrogated the induction of IFNs and ISGs, as expected, due to its essential role in the onset of cell intrinsic innate immune responses (Fig. 5B through D). However, in contrast to Huh7 and Huh7.5 cells, knockout of IRF7 had no impact on *IFNB*, *IFNL1,* or *IFIT1* induction after poly(I:C) stimulation in PH5CH cells (Fig. 5B through D). To test if the amplifying role of IRF7 became relevant at later time points after induction of its expression, the cells were re-stimulated with poly(I:C) 24 h after the initial stimulation. Yet, 6 h after this additional stimulation, no reduction of *IFIT1* or IFN induction was observed in IRF7-KO PH5CH compared to naïve cells (Fig. 5C and D).

Based on the lack of importance of IRF7 in PH5CH cells, we next asked whether increased expression of IRF3 could compensate for the IRF7 deficiency in Huh7-based models, and therefore ectopically expressed IRF3 in Huh7.5-TLR3-RIG-I cells by lentiviral transduction (Fig. 5E). Interestingly, ectopic expression of IRF3 increased *IFNB* and *IFIT1* induction after SN feeding of poly(I:C) to the level of TFX (Fig. 5F and G). This result indicated that IRF3 expression was a limiting factor for the induction of *IFNB* after SN delivery of dsRNA. However, increased IRF3 expression had no impact on *IFNB* and *IFIT1* induction after poly(I:C) TFX (Fig. 5F and G), which remained 10- to 100-fold lower than in cells reconstituted with IRF7 (Fig. 4E).

Overall, these data suggested that the function of IRF7 in *IFNB* induction was not identical in all cells of hepatic origin. While IRF3 was sufficient to mount a full response in PH5CH, IRF7 was indispensable for efficient *IFNB* induction in Huh7.5 cells. Still, IRF3 expression levels were a limiting factor for the TLR3 response in Huh7.5 cells after SN delivery of poly(I:C).

### Importance of IRF7 for IFN-β induction by Sendai virus infection

After showing that IRF7 expression restored IFN-β induction upon poly(I:C) stimulation, we analyzed its relevance in the context of more physiological conditions, using various viruses as triggers. We first used SeV as a standard model for RIG-I-mediated ISG induction. To obtain robust sensing of viral replication, we established various cell lines based on the Huh7.5-TLR3 model, ectopically expressing those PRRs that Huh7.5 cells are natively lacking (Fig. S2). We limited the number of ectopically expressed genes to the PRRs responsible for sensing the individual viruses and therefore added RIG-I for SeV (39).

**Fig 6:**
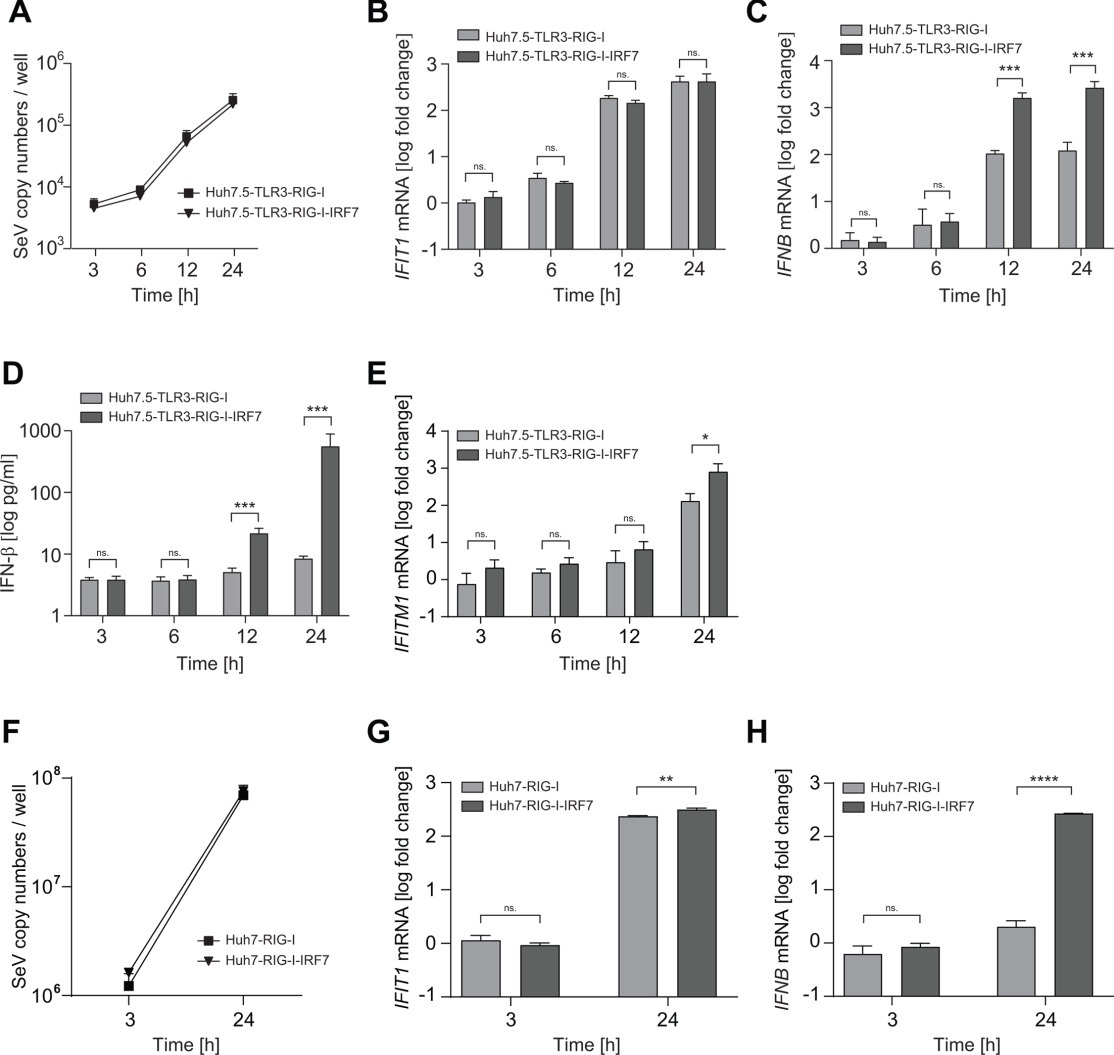
Impact of IRF7 expression on the innate immune response to Sendai virus infection in Huh7.5 and Huh7 cells. (A–E) Huh7.5 cells expressing TLR3 and RIG-I in the presence or absence of IRF7 were infected with Sendai virus at an MOI = 10. (A–C, E) At the indicated time points, total RNA was extracted and levels of (**A**) Sendai virus copy numbers, (**B**) *IFIT1*, (**C**) *IFNB,* or (**E**) *IFITM1* mRNA were determined using RT-qPCR. Data are normalized to *GAPDH* and shown as fold relative to the untreated native cell. Data represent mean values and SD of technical triplicates from four biological replicates. (**D**) The supernatant of four biological replicates was harvested and analyzed in technical duplicates by an ELISA detecting IFN-β. (F–H) Huh7 cells reconstituted with RIG-I in the presence or absence of IRF7 were infected with Sendai virus at an MOI = 10. At the indicated time points, total RNA was extracted, and levels of (**F**) Sendai virus copy numbers, (**G**) *IFIT1,* or (**H**) *IFNB* mRNA were determined using RT-qPCR. Data are normalized to *GAPDH* and shown as fold relative to the untreated native cell. Data represent mean values and SD of technical triplicates from three biological replicates.

SeV replicated efficiently in Huh7.5-TLR3-RIG-I cells in the presence and absence of IRF7 (Fig. 6A) and induced a similarly strong *IFIT1* expression in both cell lines (Fig. 6B). However, *IFNB* mRNA levels were strongly increased in cells expressing IRF7 (Fig. 6C). Importantly, secretion of IFN-β was fully dependent on the presence of IRF7 (Fig. 6D). Expression of *IFITM1* mRNA, an ISG which is dependent on JAK/STAT signaling (40), was delayed and significantly increased by IRF7 (Fig. 6E), following the kinetics of IFN-β secretion. Residual induction of *IFITM1* in cells lacking IRF7 might have been driven by type III IFNs, which were less dependent on IRF7 (Fig. S3F). Since Huh7.5 cells, beyond functional RIG-I deficiency, lack expression of RIPLET (41), a ubiquitin ligase positively regulating RIG-I responses, we analyzed the ISG induction upon SeV infection in Huh7-RIG-I cells in the presence and absence of ectopic IRF7. However, as in the case of Huh7.5, the presence of IRF7 had no impact on SeV replication (Fig. 6F) and only slightly but significantly increased *IFIT1* induction, but dramatically increased *IFNB* mRNA levels by ca. 100-fold, suggesting that Huh7.5 reconstituted with relevant PRRs can serve as a valid model for ISG induction upon virus infection.

Overall, IRF7 enhanced the otherwise limited level of *IFNB* induction in Huh7.5 and Huh7 cells not only upon artificial poly(I:C) stimulation but also in the context of viral infection.

### Impact of IRF7 on IFN-β induction by HAV, HCV, and HDV

While SeV replicates in a variety of cell lines, the comparison of the innate immune induction is often limited by the available cell culture models being either not permissive to infection with the viruses of interest or not representing the innate immune induction in physiological cells. PH5CH cells, for example, are a robust model for innate immune induction but, in our hands, not permissive for hepatotropic viruses like HAV and HCV (data not shown). Huh7.5 cells, in contrast, are permissive for most hepatotropic viruses but showed a limited innate immune response in part due to expression of a dominant negative RIG-I mutant (19). However, restoration of the RIG-I response still did not rescue *IFNB* induction upon virus infection. We therefore next aimed to see how IRF7 would impact the overall innate immune response to HAV, HCV, and HDV infection. We excluded hepatitis B virus (HBV), due to its general lack of innate immune induction (42, 43), and hepatitis E virus (HEV), due to its limited replication competence in Huh7.5 cells (data not shown).

**Fig 7:**
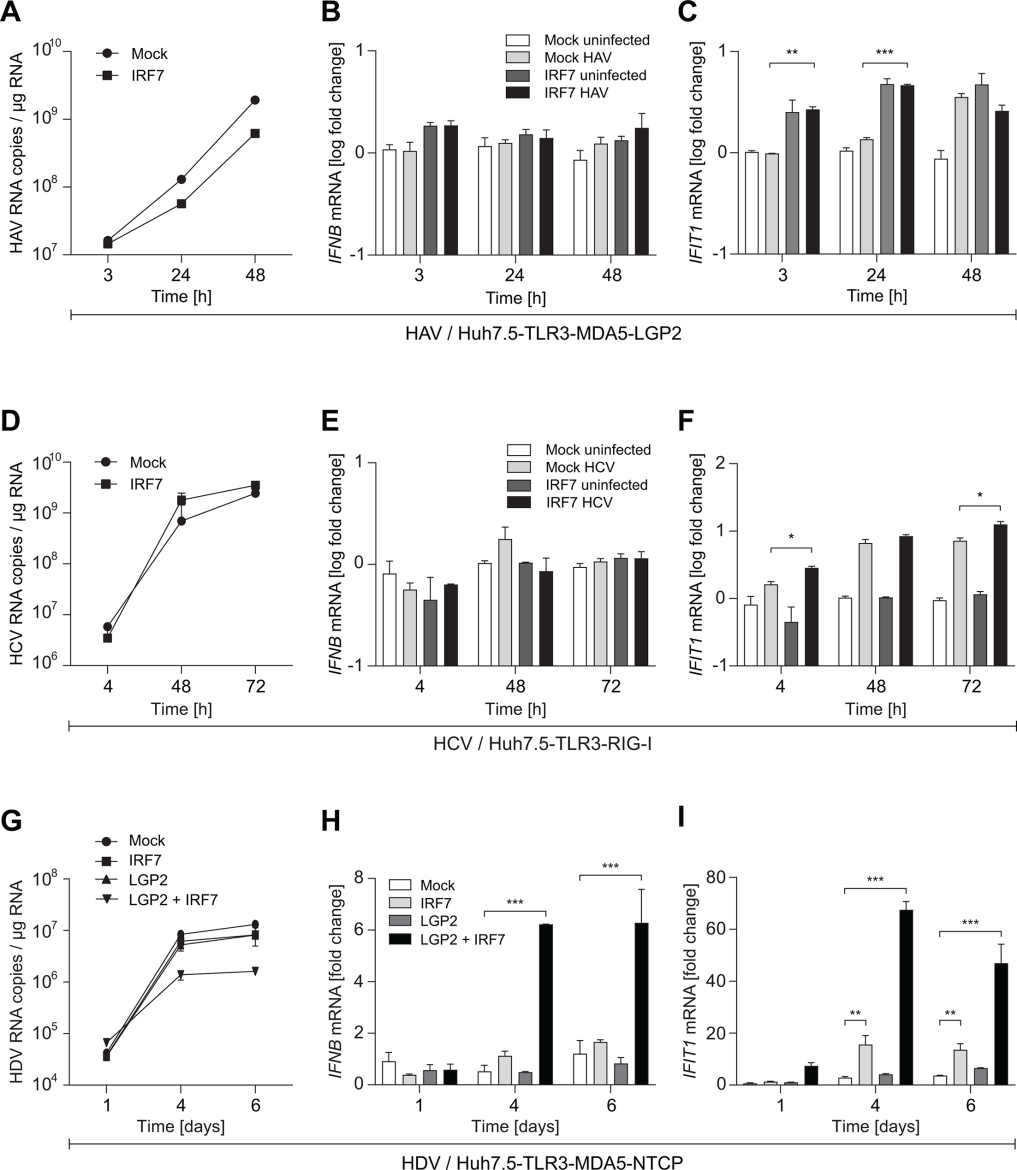
Impact of IRF7 expression on the IFN response after HAV, HCV, and HDV infection in Huh7.5-derived cells. Huh7.5 cells ectopically expressing TLR3 and MDA5/LGP2 (A–C), RIG-I (D–F), or MDA5±LGP2, as indicated (G–I), with or without additional expression of IRF7, were infected with HAV at an MOI = 1.5 (A–C), HCV at an MOI = 1 (D–F), or HDV at an MOI = 1. At the indicated time points, total RNA was extracted, and levels of viral copy numbers (**A, D, G**), *IFNB* (**B, E, H**), and *IFIT1* mRNA (**C, F, I**) were determined using RT-qPCR. ISG data are normalized to *GAPDH* and shown as fold relative to uninfected cells. Note that for HDV, only data from infected cells are shown in panels G–I, but normalization was done relative to uninfected cells as well. All data represent mean values and SD of technical triplicates from biological duplicates.

To study HAV-mediated innate immune induction, we used Huh7.5-TLR3-MDA5-LGP2 cells, since MDA5 and LGP2 have been shown to be essential for HAV sensing (26, 44). Both receptors were ectopically expressed by lentiviral transduction, since expression levels in Huh7 and Huh7.5 cells were much lower than in PHH (Tables S5 to S9; IFIH1 corresponds to MDA5). HAV replication was slightly reduced by IRF7 expression (Fig. 7A), but here, only marginal *IFIT1* induction was found, which was slightly triggered by IRF7 expression, even in the absence of HAV (Fig. 7C) and no *IFNB* induction at all (Fig. 7B).

For HCV, our former studies identified TLR3 as the main dsRNA sensor (25). RIG-I was additionally expressed, despite potent MAVS cleavage by NS3/4A (45). In line with previous studies (25, 26), HCV infection mildly induced expression of *IFIT1*, which was only marginally increased by IRF7 (Fig. 7F), whereas no induction of *IFNB* mRNA was found in naïve or IRF7-expressing Huh7.5 cells (Fig. 7E). HCV replication was furthermore not affected by IRF7 (Fig. 7D).

HDV generally shows a strong innate immune response, including IFN secretion in permissive HepaRG cells and PHH, which is dependent on MDA5 (30) and requires LGP2 (46). We therefore generated Huh7.5-TLR3-MDA5 cells expressing NTCP, which is the entry receptor of HDV (28), and compared the additional impact of LGP2 and/or IRF7 on ISG induction. Interestingly, the presence of MDA5 and IRF7 was sufficient to induce *IFIT1* upon HDV infection, even in the absence of LGP2 (Fig. 7I), but a significant induction of *IFNB* essentially required LGP2 and IRF7 expression (Fig. 7H), which further increased the *IFIT1* levels (Fig. 7I). The HDV-mediated innate immune response upon IRF7 and LGP2 expression further resulted in an inhibition of HDV replication (Fig. 7G).

By reconstituting the expression of IRF7, IFN-β induction was restored in Huh7-based cells to the levels observed in PHH and PH5CH cells upon poly(I:C) stimulation. Utilizing this model for a comparative analysis of viral IFN type I induction, HCV, and HAV infection did not result in *IFNB* induction. However, upon HDV and Sendai infection of Huh7.5 cells expressing IRF7, we observed a significant induction of *IFNB*.

## DISCUSSION

This study provides a novel Huh7.5 cell-based model to investigate innate immunity upon viral infection. Our comprehensive transcriptomic analysis compared the poly(I:C) response of PHH, immortalized hepatocytes, and Huh7-based hepatocellular carcinoma cell lines. The most remarkable difference was the generally reduced IFN-β induction in Huh7-derived cell lines, which could be rescued by reconstitution of IRF7 expression, thereby enhancing the innate immune competence of Huh7-derived cells, which are a widely used model in virology research, particularly on hepatotropic viruses.

We found that Huh7 and Huh7.5 cells lacked expression of IRF7. Given the pivotal role of IRF7 in cancer development and suppression (reviewed in reference 47), this is likely due to the tumor origin of the cells. IRF7 suppression by promoter methylation is found in many HCC samples (48), where Huh7 cells were initially established from reference 21. In line with the role of IRF7 as a master regulator of type I IFN (4, 5, 11), we observed a robust increase in the otherwise weak IFN-β induction after reconstituting IRF7 expression in Huh7 and 7.5 cells. This brought the cell intrinsic immune response of Huh7-based models to the levels observed in PHH, solving one of the fundamental flaws of this virus infection model. Even though IRF7 and IRF3 have overlapping roles in the induction of IFN-β, interestingly, ectopic expression of IRF3 in Huh7.5 cells did not increase the weak IFN-β induction upon TFX of poly(I:C), but merely brought up the induction upon SN stimulation to the same level. These data suggest that the IRF3 expression level determines the strength of stimuli needed to reach maximum PRR response. This can potentially be explained by the distinct function of IRF3 and IRF7. Since IRF3 is thought to be most important for early phase induction of type I IFN (49), the higher sensitivity for induction we observed can be expected. IRF7, however, is particularly important for later amplification of IFN induction (49): low levels of IRF7 are initially involved in priming of IFN expression, in turn upregulating IRF7 and generating a positive feedback loop (38). This nicely explains how IRF7 expression in Huh7-based cells drastically enhanced the otherwise limited amplitude of the peak IFN-β induction. In contrast to the prominent effect of IRF7 expression in Huh7 and Huh7.5 cells, we observed no reduction of IFN-β induction in PH5CH-IRF7 KO cells, as shown in A549 cells before (27). Potentially, the potent stimulation with poly(I:C) masked the absence of IRF7’s amplifying effect on IFN-β. The KO of IRF3, in contrast, abrogated the induction of IFN-β in PH5CH. The lack of IFN-β induction upon IRF3 KO was potentially due to the low basal levels of IRF7 in non-immune cells (10). Studies in IRF3/7-double KO MEFs showed reconstitution of either IRF3 or IRF7 was sufficient to restore IFN-β induction (49). This argues for a higher relevance of IRF3 in the PH5CH model due to differences in expression levels, rather than the function of the two factors. However, in contrast to PH5CH, higher IRF3 expression could not replace the essential role of IRF7 for IFN-β induction in Huh7.5 cells, suggesting cell type-specific differences, likely due to differential expression of other important factors of the cell intrinsic innate immune response. Given that IRF7 is expressed at low levels in most non-immune cells, stable expression of IRF7 does not display the physiological kinetics of IRF7 induction. While this needs to be considered in the interpretation of the data, the gene induction profiles in our Huh7-IRF7 model accurately matched the gene induction in PHH and PH5CH cells upon poly(I:C) stimulation and virus infection, emphasizing the value of this model.

Even though IRF7 is also involved in type III (50) IFN induction, this study focused on type I IFN, since we found comparable levels of *IFNL1* in Huh7-based cells as in PHH and PH5CH, which did not contribute to the antiviral effects of secreted cytokines found in the supernatant of stimulated Huh7 and Huh7.5 cells. However, some reports claimed that IRF7 is the determining factor for IFNL2 and IFNL3 induction (50), which were shown to be induced by HCV infection (51). Future studies comparing the induction of IFNL1-3 and their contribution to the antiviral effect of secreted cytokines will likely further reveal the specific function of IRF7 in hepatic cell lines. Regarding other type I IFNs beyond IFN-β, we found that PHH, upon poly(I:C) stimulation, did not show induction of any IFNA, nor did PH5CH (Tables S1 to S4). In general, IFNAs are primarily produced by plasmacytoid dendritic cells (52). We therefore focused on IFNB in our experiments, since our aim was to establish a cell culture model representing primary hepatic cells.

While reconstitution of IRF7 expression restored IFN-β production upon poly(I:C) stimulation to the level of PHH and PH5CH, induction by virus infection revealed more diverse results. SeV, a widely used model virus in studies of the RIG-I pathway, showed an IFN-β induction comparable to poly(I:C), which was strictly dependent on the presence of IRF7 in Huh7 and Huh7.5 cells, arguing for a gain in physiological relevance. Similar data were obtained for HDV infection, in line with literature universally demonstrating induction of type I IFN in PHH, HepG2 cells, and in NTCP transgenic mice (30, 53). In contrast, IRF7 expression had very limited impact on the cell intrinsic innate immune response induced by HAV and HCV in Huh7.5 cells. HAV infection in chimpanzees was described to induce only a minimal ISG response (54). In more recent reports, HAV was shown to robustly induce chemokines and type III IFN, but not type I IFN in PHH and HepG2 cells (44). Here, we found no ISG induction upon HAV infection in Huh7.5 cells reconstituted with appropriate PRRs and IRF7, in contrast to a clearly detectable innate immune response against HAV in HepG2 cells observed in a previous study (26). Therefore, an additional component beyond IRF7 might be required for HAV-mediated innate immune response, still missing in our model. Previous studies showed that Huh7.5 cells are missing RIPLET, an E3 ligase involved in RIG-I signaling (41, 55). While this limitation should be considered when interpreting ISG induction upon virus infection, MDA5 exclusive sensing of HAV (26, 56) should not be limited by this defect. This also applies to HCV, which is mainly sensed by TLR3 (25) and potently counteracts MAVS and RIPLET signaling (41). Generally, since we observed potent induction of ISGs upon Sendai virus infection in Huh7.5 cells and no major difference to Huh7 cells, severe effects of RIPLET on RIG-I signaling seem unlikely in our models. In addition to a defect in ISG induction, a potential low ISG induction upon HAV infection might be masked by a mild increase in ISG baseline levels that was found in Huh7.5 cells upon ectopic expression of LGP2 in a previous study (26). HCV infection, in comparison, did induce a detectable innate immune response in Huh7 cells, which was independent of IRF7 but lacked IFN-β production. HCV was shown to induce an ISG response in chimpanzees (54) and also in the liver of chronically HCV-infected patients (57). However, the data on type I IFN in physiological models are conflicting. In HCV infection of the human liver, no IFN-β was detected despite ISG induction (58). In HCV-infected primary human liver cultures, efficient induction of type III, but not type I IFNs, was found in one study (14), whereas another study reported type I IFN induction upon HCV infection in PHH (59). More recently, HCV infection of human liver chimeric mice demonstrated that ISG induction was focused on infected hepatocytes, arguing against a massive secretion of IFNs (26). Overall, whether or not HCV-infected hepatocytes induce type I IFNs might strongly depend on the experimental conditions, including the potential impact of other cell types in primary cultures and tissues. Despite the differences in the ISG response profile of Huh7 and Huh7.5 cells compared to more authentic models like PHH and PH5CH, it should be noted that Huh7-based cell lines are the only culture model allowing replication of all hepatitis viruses including those with otherwise limited culturing options, like HCV, HAV (17), or HEV (60), but also of HBV and HDV, upon expression of their native receptor (28). Additional expression of IRF7 will likely allow us to study the impact of the innate immune response in infected cells on viral infection more accurately. Huh7-based models have further been shown to be permissive for a variety of other viruses replicating in the liver, including severe acute respiratory syndrom coronavirus 2 (SARS-CoV-2) (61).

Besides the lack of IRF7 expression, we identified other ISGs with differential expression in PH5CH and PHH. While the level of ISG induction in Huh7.5-IRF7 cells upon virus infection and poly(I:C) stimulation argues against a fundamental role of these factors in cell intrinsic innate immunity, these limitations should be considered when interpreting data acquired from this model. In particular, CCL8, a pro-inflammatory cytokine recruiting NK and T cells by binding to receptors CCR1 and CCR5 (62), was not induced in Huh7-based cell lines, but in PH5CH and PHH. The lack of CCL8 production might need attention in co-culture models that have been used to characterize the interplay of immune cells with HCV-infected hepatocytes (63, 64). Finally, some differences can clearly be associated with immortalization, e.g., the high constitutive expression of MDK (65) in Huh7 and PH5CH cells. We further identified several genes shown to contribute to poly(I:C) uptake and TLR3 activation and expressed to a lower extent in Huh7-based models compared to PHH and PH5CH. This includes, e.g., Raftlin (RFTN1) (66), integrin subunit alpha M (ITGAM) (67), and Scavenger receptor class F member 1 (SCARF1) (68) (Tables S1 to S9). Since these factors have mainly been studied in myeloid cells, their impact on TLR3 response in hepatocytes will require further studies. Overall, the variations in TLR3 responses found upon SN delivery and transfection of poly(I:C), which is well established in the literature (15, 37), might mainly be due to the efficiency of uptake, rather than reflecting fundamental differences in the signaling pathways, since SN delivery requires clathrin-mediated endocytosis (7). This assumption is also supported by the fact that TFX of poly(I:C) was shown to not only result in a more potent and longer-lasting delivery of poly(I:C) than SN treatment, but also in a high level of co-localization with early endosomes (69). These findings present a possible explanation for the more potent response of Huh7-TLR3 cells toward TFX rather than SN stimulation with poly(I:C).

Given the comprehensive insights from this study on innate immunity in hepatic cells upon viral infection, it becomes clear that the restoration of IRF7 expression in Huh7 and Huh7.5 cells holds significant promise for future research applications. The reconstitution of IRF7 not only corrects a key limitation in the Huh7-based viral infection model by elevating *IFNB* responses to levels seen in PHH, but also underscores the crucial role of IRF7 in the immune response amplification phase. This could greatly improve the precision of viral infection simulations and the assessment of antiviral responses in hepatic cells, thus providing a more reliable platform for the study of hepatitis viruses and potentially other pathogens affecting the liver.

## Data Availability

All data supporting the findings of this study are included within the article and its supplemental material. Transcriptome data are deposited at the European Genome-Phenome Archive (https://ega-archive.org/studies/EGAS00001005147). Requests for data access should be referred directly to the Data Access Committee (https://ega-archive.org/dacs/EGAC00001002041).

## SUPPLEMENTAL MATERIAL

Supplemental figures jvi.00703-25-s0001.pdf Figures S1 to S4; legends for Tables S1 to S9.

Table S1 jvi.00703-25-s0002.xlsx PHH stimulated by poly(I:C) transfection.

Table S2 jvi.00703-25-s0003.xlsx PHH stimulated by poly(I:C) supernatant feeding.

Table S3 jvi.00703-25-s0004.xlsx PH5CH stimulated by poly(I:C) transfection.

Table S4 jvi.00703-25-s0005.xlsx PH5CH stimulated by poly(I:C) supernatant feeding.

Table S5 jvi.00703-25-s0006.xlsx Huh7-TLR3 stimulated by poly(I:C) transfection.

Table S6 jvi.00703-25-s0007.xlsx Huh7-TLR3 stimulated by poly(I:C) supernatant feeding.

Table S7 jvi.00703-25-s0008.xlsx Huh7.5-TLR3 stimulated by poly(I:C) transfection.

Table S8 jvi.00703-25-s0009.xlsx Huh7.5-TLR3 stimulated by poly(I:C) supernatant feeding.

Table S9 jvi.00703-25-s0010.xlsx Huh7.5-RIG-I stimulated by poly(I:C) transfection.

ASM does not own the copyrights to Supplemental Material that may be linked to, or accessed through, an article. The authors have granted ASM a non-exclusive, world-wide license to publish the Supplemental Material files. Please contact the corresponding author directly for reuse.
