# Supplementary material for: Reconstitution of interferon regulatory factor 7 expression restores interferon beta induction in Huh7 cells
Source: J Virol. 2025 May 23;99(6):e00703-25. doi: 10.1128/jvi.00703-25 (PMC12172482; doi:10.1128/jvi.00703-25)
Supplement: Supplemental figures — Figures S1 to S4; legends for Tables S1 to S9. [file jvi.00703-25-s0001.pdf]

Supplementary Materials to the manuscript:

Reconstitution of interferon regulatory factor 7 expression restores interferon beta induction in Huh7 cells

Andreas Betz, Hao-En Huang, Zuguang Gu, Ombretta Colasanti, Teng-Feng Li, Jasper Hesebeck-Brinckmann, Nadine Gillich, Gnimah Eva Gnouamozi, Matthias Schlesner, Florian W. R. Vondran, Stephan Urban, Ralf Bartenschlager, Marco Binder, Volker Lohmann

Supplementary Figures 1-4

Legends to supplementary tables 1-9

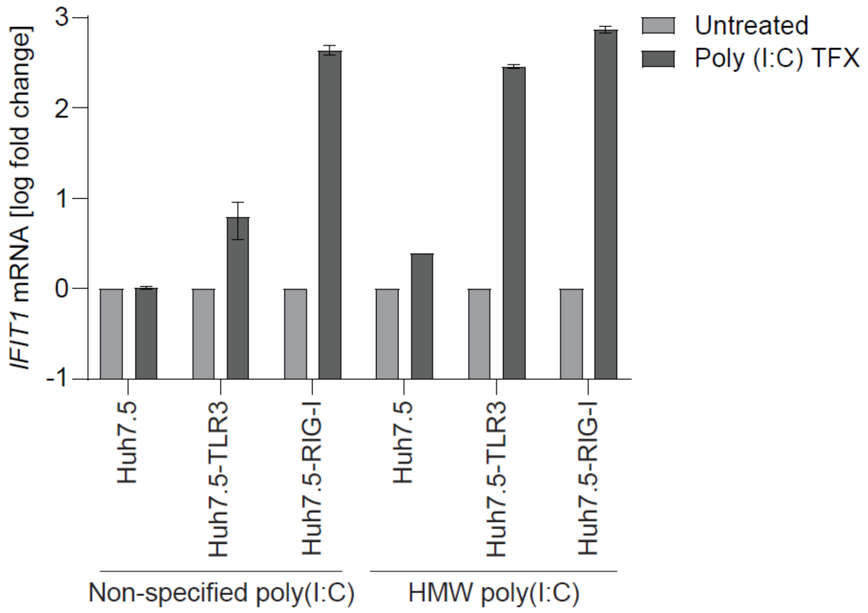

Figure S1: Comparison of different poly(I:C) qualities in Huh7.5 cells. Naïve Huh7.5 cells and Huh7.5 with either reconstituted TLR3 or RIG-I as indicated were transfected with either 0.5 µg poly(I:C) with undefined molecular weight (Sigma-Aldrich) or high-molecular-weight (HMW) poly(I:C) (Invivogen). *IFIT1* mRNA expression was determined by RT-qPCR, normalized to GAPDH and expressed as fold change [log fc] relative to the respective untreated cells (light grey). Mean values and SD from 3 biological replicates.

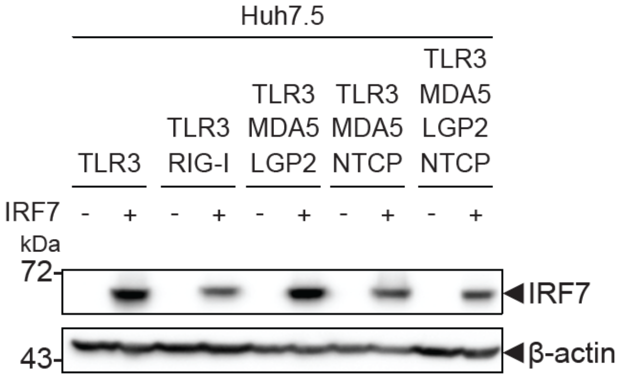

Figure S2: Ectopic expression of IRF7 in Huh7.5 cell variants. Huh7.5 cells ectopically expressing the indicated genes, were transduced with lentiviral vectors encoding *IRF7*. IRF7 expression was determined by Western Blot and compared to non-transduced cells.

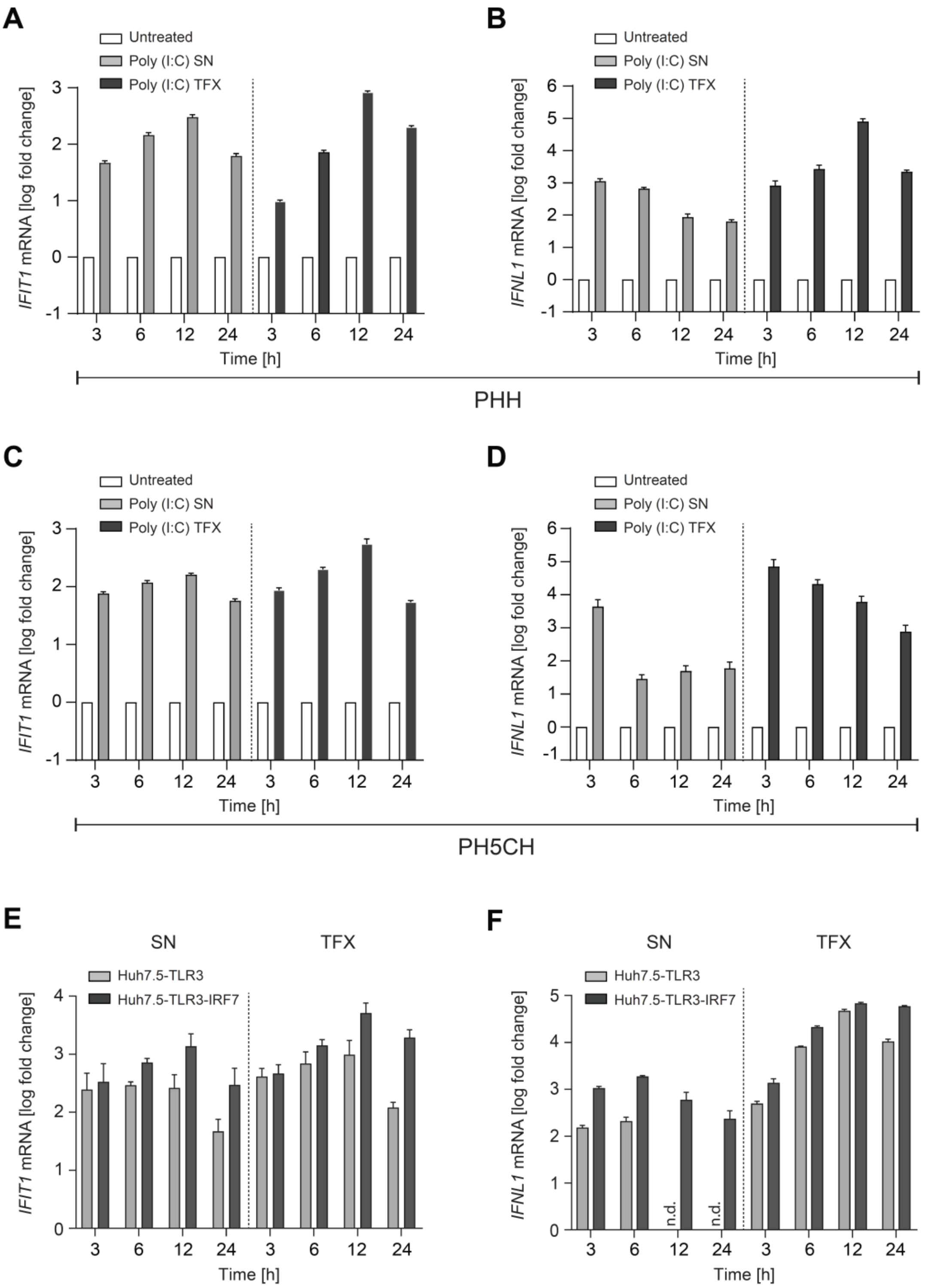

Figure S3: *IFIT1* and *IFNL1* induction upon poly (I:C) stimulation. Indicated cells were stimulated with poly(I:C) either delivered to the supernatant (10 µg/ml) or by transfection (0.5 µg/ml). At the indicated time points, total RNA was extracted and mRNA levels of *IFIT1* (A, C, E), or *IFNL1* (B, D, F) were determined using RT-qPCR. Data are normalized to *GAPDH* and shown as fold relative to the untreated naive cells. Data represent mean values and SD of technical triplicates from two biological replicates (E, F), or one representative experiment (A-D).

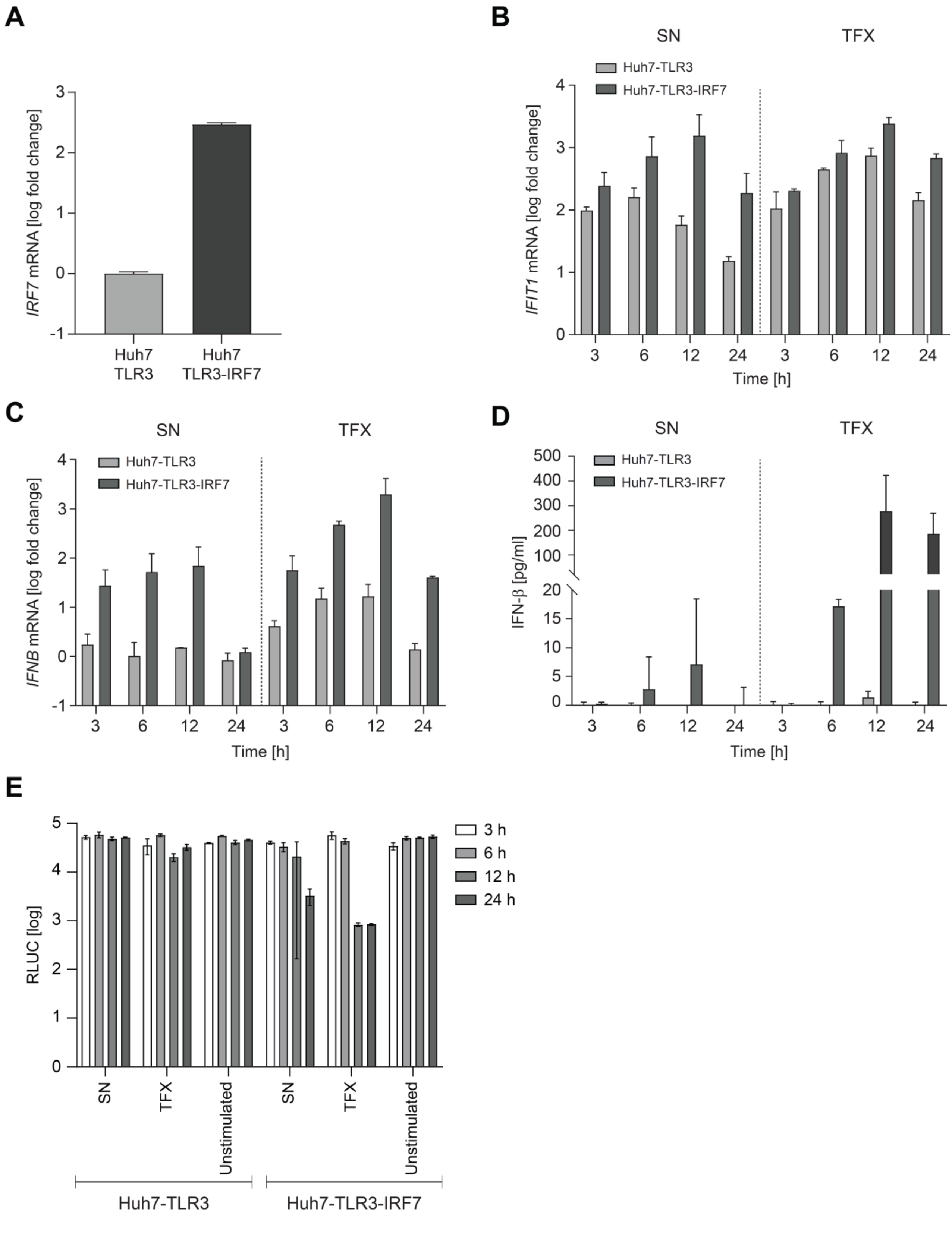

Figure S4: Impact of *IRF7* expression on innate immune response in Huh7 cells. (A) Huh7 cells were transduced with lentiviral vectors encoding *TLR3* and *IRF7*, as indicated. The level of *IRF7* mRNA was quantified using RT-qPCR. *IRF7* expression was normalized to *GAPDH* and shown as fold relative to the Huh7-TLR3

cells. (B-E) Huh7-TLR3 cells with or without IRF7 expression were stimulated with poly(I:C) either delivered to the supernatant (SN: 10 µg/ml) or by transfection (TFX: 0.5 µg/ml). At the indicated time points, total RNA was extracted, and mRNA levels of *IFIT1* (B) and *IFNB* (C) were determined using RT-qPCR. Data are normalized to *GAPDH* and shown as fold relative to the untreated Huh7.5-TLR3 cells. In addition, secreted IFN-β was quantified in cell supernatant by ELISA in technical duplicates (D) and biological activity of secreted IFNs was determined using indicator cells (E). Supernatants from indicated cells and conditions were harvested at different time points after poly(I:C) or mock (untreated) stimulation and transferred to Huh7 cells containing a persistent HCV reporter replicon, encoding Firefly luciferase (1). Luciferase activity was determined in cell lysates 72h after supernatant transfer and is shown in relative light units (RLU). Reduced RLU counts indicate inhibition of viral replication by secreted IFNs. Data represent mean values and SD of technical triplicates from two biological replicates (A-C). The supernatant of two biological replicates was harvested and analyzed in technical duplicates by an ELISA detecting IFN-β and in technical triplicates for luciferase activity.

References to supplementary figure legends

1. Vrolijk JM, Kaul A, Hansen BE, Lohmann V, Haagmans BL, Schalm SW, Bartenschlager R. 2003. A replicon-based bioassay for the measurement of interferons in patients with chronic hepatitis C. J Virol Methods 110:201-9.

Table S1-S9: Normalized raw counts and pairwise differential expression analysis of all NGS datasets included in the study. Each stimulated dataset is compared to its untreated control. Note that all analyses are based on biological triplicate samples, except for PHH, which are based on individual sets from four different donors.

Table S1: PHH stimulated by poly(I:C) transfection. Normalized raw counts and pairwise differential expression analysis of stimulated cells compared to untreated controls.

Table S2: PHH stimulated by poly(I:C) supernatant feeding. Normalized raw counts and pairwise differential expression analysis of stimulated cells compared to untreated controls.

Table S3: PH5CH stimulated by poly(I:C) transfection. Normalized raw counts and pairwise differential expression analysis of stimulated cells compared to untreated controls.

Table S4: PH5CH stimulated by poly(I:C) supernatant feeding. Normalized raw counts and pairwise differential expression analysis of stimulated cells compared to untreated controls.

Table S5: Huh7-TLR3 stimulated by poly(I:C) transfection. Normalized raw counts and pairwise differential expression analysis of stimulated cells compared to untreated controls.

Table S6: Huh7-TLR3 stimulated by poly(I:C) supernatant feeding. Normalized raw counts and pairwise differential expression analysis of stimulated cells compared to untreated controls.

Table S7: Huh7.5-TLR3 stimulated by poly(I:C) transfection. Normalized raw counts and pairwise differential expression analysis of stimulated cells compared to untreated controls.

Table S8: Huh7.5-TLR3 stimulated by poly(I:C) supernatant feeding. Normalized raw counts and pairwise differential expression analysis of stimulated cells compared to untreated controls.

Table S9: Huh7.5-RIG-I stimulated by poly(I:C) transfection. Normalized raw counts and pairwise differential expression analysis of stimulated cells compared to untreated controls.
